# Recent HIV infection among newly diagnosed cases and associated factors in the Amhara regional state, Northern Ethiopia: HIV case surveillance data analysis (2019-2021)

**DOI:** 10.3389/fpubh.2022.922385

**Published:** 2022-11-15

**Authors:** Tefera Alemu, Misganaw Ayalew, Mahteme Haile, Abraham Amsalu, Alie Ayal, Fisseha Wale, Amogne Belay, Birhanu Desta, Tesfahun Taddege, Damtie Lankir, Belay Bezabih

**Affiliations:** ^1^Amhara NRS Public Health Institute, Public Health Emergency Management Directorate, Bahir Dar, Ethiopia; ^2^ICAP in Ethiopia, Amhara Regional Office, Bahir Dar, Ethiopia; ^3^House of Peoples' Representatives of FDRE, Addis Ababa, Ethiopia

**Keywords:** HIV case surveillance, recent HIV infection, HIV hotspot, Amhara, Ethiopia

## Abstract

**Background:**

Distinguishing a recent from long-standing HIV infection is a critical step to reduce new infections in 2030. Therefore, this analysis determines the proportion of recent HIV infections among newly diagnosed cases and associated factors in the Amhara regional state between 2019 and 2021.

**Methods:**

We got the HIV case-based surveillance dataset (July 2019 up to August 12/2021) from the Amhara Public Health Institute. Recent infection is an infection gained within the last 12 months as identified by Asante recency test kits. Logistic regression was carried out to identify factors associated with recent infection. Adjusted odd with 95% CI and a *p*-value of < 0.05 was considered to declare significant associations.

**Results:**

Out of 5,689 eligible cases, 3,129 (55%) recency tests had been performed. The proportion of recent HIV infection is 443 (14.2%, 95% CI: 13, 15.4%). High proportion of recent infections is reported from Bahir Dar city (23.3%), Central Gondar (17.7%), West Gojjam (16.5%), North Shewa (16.5%) and South Gondar zones (15.7%). Besides, the proportion of recent infection is high among clients aged ≥ 51 years (32.4%), illicit drug users (30.6 %), homelessness (28.5%), current commercial sex workers (27.9%), prisoners (21.1%), and among clients with invasive medical procedures (22.2%). Recent infection is significantly associated with females (AOR: 1.9, 95% CI: 1.2–3.1), secondary and above education (AOR: 2.1, 95% CI: 1.3–3.4), commercial sex workers (AOR: 1.8, 95% CI: 1.2–2.7), having contact with index case (AOR: 0.5, 95% CI: 0.3–0.8) and illicit drug utilization (AOR: 3.6, 95% CI: 1.1–12.4).

**Conclusion:**

In the Amhara region, the proportion of recent HIV infection is high with marked variation across sociodemographic characteristics. We identified the risk or preventive factors associated with a recent infection. Therefore, all HIV responders should target their prevention efforts toward hot spot areas and sub-populations to stop further transmission.

## Introduction

Ethiopia has made significant progress toward universal coverage of HIV diagnosis, treatment and viral suppression of persons living with HIV. For instance, in the past two decades, Ethiopia has been successful in reducing the HIV prevalence rate from 3.3 % in 2000 to 0.9 % in 2017 and in decreasing AIDS-related deaths from 83,000 deaths in 2000 to 15,600 in 2017 (1). However, the existing HIV/AID information system is suffering from several limitations. For instance, it doesn't distinguish whether the diagnosis of a new HIV case is because of an increase in HIV transmission or an increased testing coverage for undiagnosed infections, lacks real-time data reporting system and doesn't track individual-level data. Strengthening the national HIV/AIDS strategic information systems through longitudinal and individual-level data is the World Health Organization recommendation to its member states to better understand subnational epidemics and guide more focused interventions (2, 3). Thus to overcome the above limitations, a new surveillance approach is started in Ethiopia in 2018/19 that emphasizes distinguishing recent from long-standing HIV-1 infections among newly identified HIV cases (4). The surveillance case definition for recent infection is an infection acquired within the last 12 months as identified by using Asante HIV recency test kits (4).

People with recent infections have a high viral load, immature and weak immune response, continued high-risk behaviors, high probability of ongoing transmission, and high possibilities to remember their sexual contacts (4, 5). As a result, the test offers new opportunities to focus resources on geographical areas and sub-populations with a high number of recent infections, to assess the risk behaviors, to improve the health of individuals living with HIV and speed up epidemic control (5–8).

Therefore, analysis of any data is the backbone of interpreting any public health raw data; and as being in the public health domain, the HIV data also needs to be interpreted as of other data as well; since it is one of the public health concerns in Ethiopia. Even though the national HIV case-based surveillance system is at its infancy stage, the existing data is not yet analyzed and presented to decision-makers and the scientific community so far. Therefore, this analysis determines the proportion of recent HIV-1 infection among newly diagnosed HIV cases and to identify factors that contribute to recent infections in the Amhara regional state, Northern Ethiopia.

## Methods and materials

### Data sources and extraction

In this analysis, we got data from the Amhara Public Health Institute HIV case-based surveillance database/RedCap server. The analysis covered data reported from July 2019 up to August 12/2021. Trained health care workers at HIV testing outlets are collecting the data using a Case-Based Report Form (CRF) for public health surveillance. Data clerks encode the surveillance data into a central server using a web-based application called RedCap within 14 days of HIV diagnosis.

### Study setting and population

**Figure 1:**
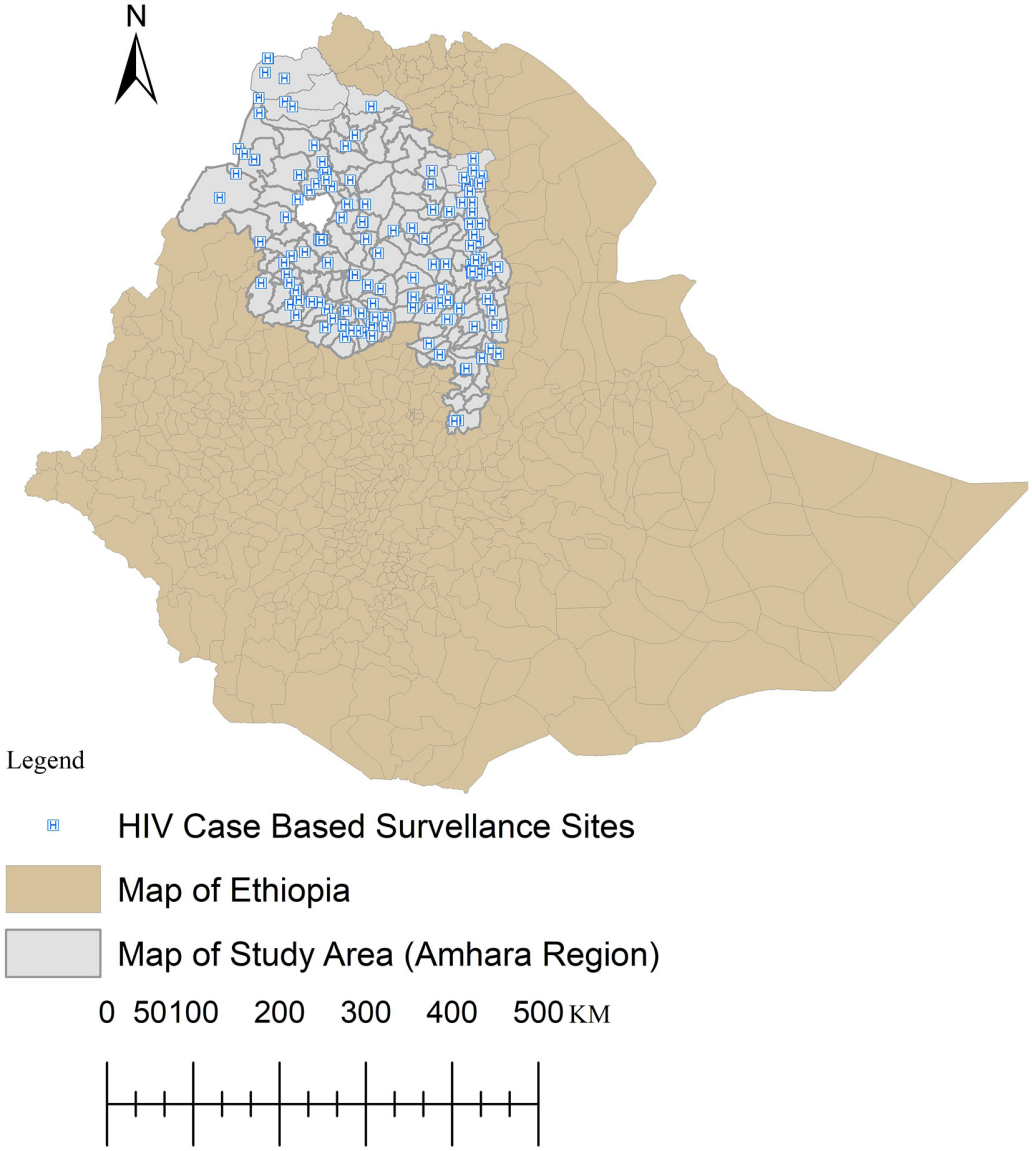
Distribution of HIV case-based surveillance sites in the study area, northern Ethiopia.

In July 2019, the Amhara Public Health Institute with support from International Center for AIDS Care and Treatment Program (I-CAP Ethiopia) and the Center for Disease Prevention and Control (CDC) has endorsed a new surveillance approach called *HIV Case Surveillance*. As shown in Figure 1 below, 130 health facilities are enrolled in the system in a scale-up approach and they report data through an online and real-time reporting software called RedCap (Research data Capturing). These health facilities are currently reporting about 90% of the regional HIV cases. Currently, the Amhara region is estimated to have over 24,769,157 people (Figure 1). For this analysis, there was no sampling, rather we used the universe of the records between July 2019 and August 12/2021.

### Inclusion criteria

The target population for Asante recency testing is clients ≥15 years old. So, we excluded below 15 years old clients from the analysis.

### Asante HIV-1 rapid recency assay

It is a point-of-care recency test (POC-RT) that combines verification of HIV diagnosis and detection of recent HIV-1 infection in one testing device and is now available as a commercial kit (Asante recency test^®^, Sedia Biosciences, Beaverton, Oregon, USA). The test can be completed rapidly in 20 min. The test has three lines, a Control line (C line), an HIV diagnostic verification line (V line), and a third line [Long-term (LT) line], which distinguishes recent infections from long-term infections. The presence of only the control line (C line) shows HIV-negative status, while two lines (C and V lines) shows recent infection, and all three lines (C, V, and LT lines) shows long-term infection (9). Recency test targets are those clients ≥15 years of age with HIV-1 infection only. As reported by the US CDC, the Asante recency test kit has a high diagnostic sensitivity (>99%) and specificity (> 98%) (10).

The test strip comprises several materials which in combination can detect HIV antibodies when a blood, serum or plasma sample containing HIV antibodies is added to the sample buffer tube. The recency assay uses the principle of avidity, binding strength of the HIV-1 antibodies present in an infected individual's blood. Following HIV-1 infection, the immune system produces low avidity HIV-1 antibodies early in the infection, and as time runs, the immune system matures and produces high avidity HIV-1 antibodies (11).

### Data management and statistical analysis

We exported data from the online RedCap server to SPSS 21 for analysis. After data cleaning and coding, we computed descriptive measures to describe the study participants and the outcome variable. We classified the outcome variable as recent and long-term infection. The Hosmer-Lemeshow goodness of fit test indicated that our regression model is a good fit for the data (α = 0.87). Binary logistic regression analyses have been carried out to identify factors associated with recent HIV infection. Variables with a *P*-value of ≤0.2 in simple binary logistic regression analysis are considered in the multivariable logistic regression analysis model to control for confounding. The backward likelihood ratio method was used for variable selection procedures. A *P*-value of <0.05 was considered a statistically significant association. Crude and adjusted odds ratios with a 95% confidence interval were computed to observe the strength of association between the outcome variable and independent variables.

### Operational definitions

#### HIV case surveillance

HIV case surveillance is a systematic, ongoing and longitudinal reporting of data on newly diagnosed HIV cases and their subsequent sentinel events to a public health authority (4). However, at current stage, the system captures individual level data acquired only with 14 days of HIV diagnosis and we call it HIV case reporting surveillance.

#### A newly identified HIV case

An individual who has a confirmed diagnosis of HIV infection that was not diagnosed in the past (4) using a national testing algorithm (12).

#### Recent HIV-1 infection

This is an infection that was likely acquired within 12 months preceding recency test date as identified by using Asante HIV recency test kits. Because of the lack of routine viral load testing, we took the probably recent infection as a final results. The target population for HIV recency testing surveillance is limited to adults ≥15 years of age (4).

#### Long term infection

Long Term Infection is HIV infection which is likely acquired before 12 months of the recency test date.

#### Invasive medical procedure

Is a purposeful entry to the body, usually by incision or percutaneous puncturing or by inserting instruments into the body openings for medical reasons and that might pose an additional risk of HIV infection. Examples are blood transfusion and needle injections (4).

HIV Index Case: Is a person who initially is diagnosed to have HIV infection and will serve as a contact person to identify and investigate his/her sexual/biological contacts and risk network.

## Ethical approval and consent for publication

Ethical approval was obtained from the Amhara Public Health Institute Ethical Review Committee (Protocol No: H/R/T/T/D/5/3). Also, a permission letter to analyze and publish the data is obtained from the respective bodies of the institute. At the time of HIV diagnosis, informed consent from each client is being obtained. The Ethiopian Public Health Institute prepared nationally and already approved the contents of consent as part of the national HIV surveillance guidelines. We conducted the study under the Declaration of Helsinki.

## Results

### Sociodemographic characteristics of participants

In the study period, 6,122 newly identified HIV cases have been reported to the region from 130 health facilities. From these, 5,689 (92.9%) cases were eligible for HIV recency test. But, only 3,150 (55.4%) recency tests have been performed and 3,129 (99.3%) results were valid and the rest 21 results were inconclusive. The chief reason behind missed recency testing is COVID 19, but also test kit stock out and lack of attention contributes a few. Therefore, we included those clients with valid recency test results (3,129) in the analysis as study participants.

The mean age of clients at HIV diagnosis was 34(±10 SD) years, ranging from 15 up to 84 years; but the majority (72.3%) is in between 21 and 40 years. Two-third (66.2%) of them are females and 72.1% are living in urban areas. About, 61.6% of the clients are not married/cohabiting at the time of HIV diagnosis. As reported by clients, at the time of HIV diagnosis, 33.2% of them were working as a casual laborer, 16% were farmers, 14.3% were current commercial sex workers, while 31.5% of the clients had a history of commercial sex practice in the past 12 months of the respective years. In addition, 83.5% of the clients are living in houses/apartments and 43.3% have no formal education (Table 1).

**Table 1:** Socio-demographic characteristics of recently infected HIV clients in the Amhara regional state, northern Ethiopia, 2021 (*N* = 3,129).

**Variables**	**Category**	**Recency test results**		**Total (*N*, %)**
		**Recent (*N*, %)**	**Long term (*N*, %)**	
Age in years (*N =* 3,129)	15–20	9 (4.7)	182 (95.3)	191 (6.1)
	21–25	53 (10.6)	445 (89.4)	498 (15.9)
	26–30	82 (11.1)	656 (88.9)	738 (23.6)
	31–35	76 (13.6)	482 (86.4)	558 (17.8)
	36–40	88 (18.8)	379 (81.2)	467 (14.9)
	41–45	46 (16.4)	235 (83.6)	281 (9.0)
	46–50	30 (14.0)	184 (86)	214 (6.8)
	≥51	59 (32.4)	123 (67.6)	182 (5.8)
Sex (*N =* 3,129)	Male	115 (10.9)	1,742 (84.2)	1,059 (33.8)
	Female	328 (15.8)	944 (89.1)	2,070 (66.2)
Residency (*N =* 3,129)	Urban	329 (14.6)	1,927 (85.4)	2,256 (72.1)
	Rural	114 (13.1)	759 (86.9)	873 (27.9)
Type of current residency (*N =* 3,107)	Homeless	73 (28.5)	183 (71.5)	256 (8.2)
	Shelter	23 (11.7)	173 (88.3)	196 (6.3)
	Prison	4 (21.1)	15 (78.9)	19 (0.6)
	Others	6 (14.6)	35 (85.4)	41 (1.3)
	House/apartment	336 (12.9)	2,259 (87.1)	2,595 (83.5)
Marital status (*N =* 3,105)	Not married/cohabiting	301 (15.7)	1,613 (84.3)	1,914 (61.6)
	Married/cohabiting	138 (11.6)	1,053 (88.4)	1,191 (38.4)
Current occupation (*N =* 3,069)	Commercial sex workers	122 (27.9)	316 (72.1)	438 (14.3)
	Casual Laborers	130 (12.8)	888 (87.2)	1,018 (33.2)
	Governmental/non-governmental organization	25 (12.3)	178 (87.7)	203 (6.6)
	Self/private business employed	52 (12.1)	378 (87.9)	430 (14.0)
	Unemployed/jobless	17 (11.2)	135 (88.8)	152 (5.0)
	Housewife	28 (9.3)	273 (90.3)	301 (9.8)
	Others	7 (19.4)	29 (80.6)	36 (1.2)
	Farmers	54 (11.0)	437 (89)	491 (16.0)
Educational status (*N =* 1,442)	No formal education	62 (9.9)	562 (90.1)	624 (43.3)
	Primary	63 (12.7)	435 (87.3)	498 (34.5)
	Secondary and above	49 (15.3)	271 (84.7)	320 (22.2)
Permanent and current residency is different	No	384 (14.3)	2,302 (85.7)	2,686 (85.8)
	Yes	59 (13.3)	384 (86.7)	443 (14.2)
Recent history of commercial sex practice (*N =* 1,959)	No	168 (12.5)	1,174 (87.5)	1,342 (68.5)
	Yes	138 (22.4)	479 (77.6)	617 (31.5)
Contact with index case (*N =* 3,093)	No	384 (15.1)	2,166 (84.9)	2,550 (82.4)
	Yes	56 (10.3)	487 (89.7)	543 (17.6)
Illicit drug use (*N =* 3,057)	No	406 (13.5)	2,602 (86.5)	3,008 (98.4)
	Yes	15 (30.6)	34 (69.4)	49 (1.6)
Invasive medical procedure (*N =* 3,103)	No	418 (13.8)	2,613 (86.2)	3,031 (97.7)
	Yes	16 (22.2)	56 (77.8)	72 (2.3)
Zone of current residency (*N =* 3,115)	Bahir Dar city	99 (23.2)	327 (76.8)	426 (13.7)
	Central Gondar	23 (17.7)	107 (82.3)	130 (4.2)
	West Gojjam	50 (16.5)	253 (83.5)	303 (9.7)
	North Shewa	16 (16.5)	81 (83.5)	97 (3.1)
	South Gondar	32 (15.7)	172 (84.3)	204 (6.5)
	Other regions	5 (15.6)	27 (84.4)	32 (1.0)
	Dessie city	5 (13.2)	33 (86.8)	38 (1.2)
	North Wollo	37 (13.1)	245 (86.9)	282 (9.1)
	North Gondar	27 (13.0)	181 (87)	208 (6.7)
	Gondar city	11 (12.5)	77 (87.5)	88 (2.8)
	East Gojjam	64 (12.1)	465 (87.9)	529 (17.0)
	Awi	12 (11.3)	94 (88.7)	106 (3.4)
	Waghimra	3 (10.7)	25 (89.3)	28 (0.9)
	Oromo zone	4 (10.0)	36 (90)	40 (1.3)
	South Wollo	47 (8.9)	484 (91.1)	531 (17.0)
	West Gondar	5 (6.8)	68 (93.2)	73 (2.3)
HIV diagnosis year	2011 E.C	7 (20.6)	27 (79.4)	34 (1.1)
	2012 E.C	236 (16.1)	1,229 (83.9)	1,465 (46.8)
	2013 E.C	200 (12.3)	1,430 (87.7)	1,630 (52.1)
Ever been tested before (*N =* 3,107)	No	287 (13.1)	1,900 (86.9)	2,187 (70.4)
	Yes	156 (17.0)	764 (83)	920 (29.6)
Detected *via* voluntary testing	No (Other outlets)	288 (14.0)	1,766 (86)	2,054 (65.6)
	Yes (at VCT)	155 (14.4)	920 (85.6)	1,075 (34.4)

### Recent HIV infection

**Figure 2:**
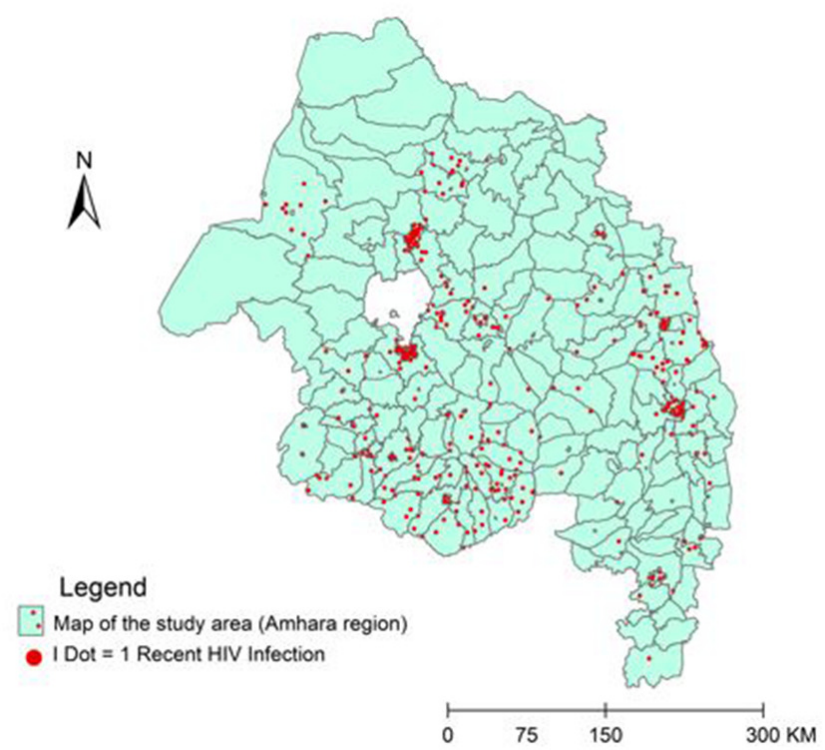
Geographic distribution of recent HIV infection in the Amhara regional state, northern Ethiopia (*N* = 443).

From 3,129 recency tests with valid results, 443 (14.2%, 95% CI: 13, 15.4%) clients are recently infected. The proportion of recent HIV infection markedly varies across zones and geographic areas (Figure 2); for instance, it is 23.2% in Bahir Dar city, 17.7% in Central Gondar zone, whereas 8.9% in South Wollo and 6.8% in West Gondar zones (Table 1).

We observed the highest proportion of recent infections among clients aged ≥ 51 years (32.4%), illicit drug users (30.6%), homelessness (28.5%) and current commercial sex workers (27.9%). The proportion of recent infection is 22.4% among clients having a recent history of commercial sex practice, 22.2% among clients having a recent history of invasive medical procedure, 21.1% in prisoners, 15.8% among females, 14.6% among urban dwellers, 15.3% in clients with secondary and above education. Besides, the proportion of recent infections is 18.8% in between 36 and 40 years old and 16.4% in the age range of 41 up to 45 years. As age increases, the proportion of recent infections also gets high (Table 1).

### Risk factors associated with recent HIV infection

In multivariable logistic regression analysis, recent HIV infection is significantly associated with sex, educational status, commercial sex practice, contact with index case and illicit drug utilization.

Accordingly, 15.8% of females are recently infected as compared to 10.9% of males (AOR: 1.9, 95% CI, 1.2–3.1). Likewise, 22.4% of clients with a recent history of commercial sex practice are recently infected as compared to the 12.5%% of their counterparts (AOR: 1.8, 95% CI: 1.2–2.7). Besides, we observed a significantly higher proportion of recent infections among illicit drug users (30.6%) as compared to 13.5% of their counterparts (AOR: 3.6, 95% CI: 1.1-12.4). Also, the odds of recent infection have become substantially increased along sides of increased educational status. Consequently, clients with secondary and above education are two times more likely to be recently infected than clients without formal education (AOR: 2.1, 95% CI: 1.3–3.4). Similarly, the opportunity to detect recently infected HIV cases is decreased by half among clients who have contact with index cases as compared to their counterparts (AOR: 0.5, 95% CI: 0.3–0.8) (Table 2).

**Table 2:** Risk factors associated with recent HIV infections in the Amhara regional state, northern Ethiopia, 2021 (*N* = 3,129).

**Study variables**	**Category**	**Recency test results**		**COR (95% CI)**	**AOR (95% CI)**
		**Recent (*N*/%)**	**Long term (*N*/%)**		
Age in years (*N =* 3,129)	21–25	53 (10.6)	445 (89.4)	2.4 (1.2–5.0)	1.2 (0.4–4)
	26–30	82 (11.1)	656 (88.9)	2.5 (1.2–5.1)	1 (0.3–3.1)
	31–35	76 (13.6)	482 (86.4)	3.2 (1.6–6.5)	0.7 (0.2–2.5)
	36–40	88 (18.8)	379 (81.2)	4.7 (2.3–9.5)	2 (0.6–6.3)
	41–45	46 (16.4)	235 (83.6)	4 (1.9–8.3)	2 (0.5–7.1)
	46–50	30 (14)	184 (86)	3.3 (1.5–7.1)	2.3 (0.2–36)
	≥51	59 (32.4)	123 (67.6)	9.7 (4.6–20.3)	5.7 (0.2–195.7)
	15–20	9 (4.7)	182 (95.3)	1	1
Sex (*N =* 3,129)	Female	328 (15.8)	1,742 (84.2)	1.5 (1.2–1.9)	1.9 (1.2–3.1)^*^
	Male	115 (10.9)	944 (89.1)	1	1
Type of current residency (*N =* 3,107)	Shelter	23 (11.7)	173 (88.3)	0.9 (0.6–1.4)	0.5 (0.6–4.8)
	Prison	4 (21.1)	15 (78.9)	1.8 (0.6–5.4)	4.8 (0.5–45.7)
	Others	6 (14.6)	35 (85.4)	1.2 (0.5–2.8)	0.5 (0.5–4.9)
	Homeless	73 (28.5)	183 (71.5)	2.7 (2.0–3.6)	0.3 (0.0–6.7)
	House/apartment	336 (12.9)	2,259 (87.1)	1	1
Marital status (*N =* 3,105)	Not married/cohabiting	301 (15.7)	1,613 (84.)	1.4 (1.2–1.8)	1.3 (0.8–2.1)
	Married/cohabiting	138 (11.6)	1,053 (88)	1	1
Current occupation (*N =* 3,033)	Casual Laborers	130 (12.8)	888 (87.2)	1.4 (0.9–2.2)	0.9 (0.4–1.9)
	Government employed	25 (12.3)	178 (87.7)	1.4 (0.8–2.4)	2.4 (0.9–6.5)
	Self/private employed	52 (12.1)	378 (87.9)	1.3 (0.8–2.2)	1.1 (0.5–2.5)
	Unemployed/jobless	17 (11.2)	135 (88.8)	1.2 (0.6–2.3)	1.1 (0.3–3.4)
	Commercial sex workers	122 (27.9)	316 (72.1)	3.8 (2.4–5.9)	0.6 (0.3–1.5)
	Farmers	54 (11)	437 (89)	1.2 (0.7–1.9)	1 (0.4–2.5)
	Housewife	28 (9.3)	273 (90.7)	1	1
Educational status (*N =* 1,442)	Primary	63 (12.7)	435 (87.3)	1.3 (0.9–1.9)	1.2 (0.7–1.9)^*^
	Secondary and above	49 (15.3)	271 (84.7)	1.6 (1.1–2.4)	2.1 (1.3–3.4)^*^
	No formal education	62 (9.9)	562 (90.1)	1	1
Recent history of commercial sex practice (*N =* 1,959)	Yes	138 (22.4)	479 (77.6)	2 (1.6–2.6)	1.8 (1.2–2.7)^*^
	No	168 (12.5)	1,174 (87.5)	1	1
Contact with index case (*N =* 3,093)	Yes	56 (10.3)	487 (89.7)	0.6 (0.5–0.9)	0.5 (0.3–0.8)^*^
	No	384 (15.1)	2,166 (84.9)	1	1
Illicit drug use (*N =* 3,057)	Yes	15 (30.6)	34 (69.4)	2.8 (1.5–5.2)	3.6 (1.1–12.4)^*^
	No	406 (13.5)	2,602 (86.5)	1	1
Invasive medical procedure (*N =* 3,103)	Yes	16 (22.2)	56 (77.8)	1.8 (1–3.1)	0.5 (0.1–4.1)
	No	418 (13.8)	2,613 (86.2)	1	1

* Significant association.

## Discussion

In the Ethiopian context, this analysis is the first of its kind about recent HIV infections among newly diagnosed HIV cases that convey valuable information for a targeted response. Therefore, we believe our findings will serve as a guide for individuals and organizations working on different aspects of the HIV program to focus their resources toward the areas and sub-populations with a high proportion of recent infections. We found the overall proportion of recent HIV infection in the region to be 14.2%. Yet, recency test is not done for a significant proportion of eligible cases (44.6%); which has the potential to alter the current findings of the study. However, the reason was largely because of the direction of the central government to stop the recency test associated with coronavirus, and absence of test kit, undocumented results, and the likes. But it is not related to the client's refusal to undergo recency testing or not related to any kind of bias.

Nevertheless, the 14.2% finding in the present study is alarming from the perspective of HIV prevention, which shows sluggish progress toward the UN AIDS strategy to reduce new infections fewer than 200,000 in 2030 (13). It is also obvious that such findings show an active HIV transmission and prevalent risky behaviors in the study area. Besides, the finding illustrates gaps in implementing the national HIV prevention strategies across the region. The proportion of recent infections markedly varies across zones; for instance, in Bahir Dar city it is 23.2%, whereas 12.5% in Gondar city. This finding indicates more transmission clusters and risky sexual networks in towns that serve as a source of infections. Therefore, this study revealed priority geographic areas and sub-populations where HIV is actively transmitting. Thus, as stated in the national HIV surveillance guideline, the enhances response strategy is case and cluster investigation to identify risky sexual networks and HIV transmission clusters (4). During the investigation, HIV status of individuals in the network and the type of risky behavior will be determined and those positive individuals should be rapidly started on treatment. These enables us to realize the individual and public health benefits of viral load suppressions, to the extent of no HIV transmission from clients with undetectable viral load (14). It is also important to provide a combination of HIV prevention services for HIV negative individuals in the sexual network like Pre-Exposure Prophylaxis for female sex workers, Self-Test, retest and risk reduction service based on the identified risky behavior. *Enhanced Response* efforts to recent infections and/or cases with risk factors/group can be maximized through a strong coordination platform like a Public Health Emergency Operation Center. Nonetheless, the 14.2% finding in the present study is lower than the 30.4% finding in Germany (15), the 19% report from Singapore (16) and the 17% report in Uganda (17), but it is higher than the 9.5% finding from northern China (18), the 8.6% finding in Kenya (19), the 6.1% finding from Rwanda (20) and the 3% finding in Malawi (21). This difference comes from real variation in HIV incidence in the respective countries or difference in recent infection testing algorisms.

We identified important risk factors associated with recent HIV infection that has to be considered to guide the prevention efforts of HIV responders. The first variable is being a female, which increases the likelihood of recent infection with over two folds as compared to males, which is also reported by WHO regional office for Africa (2), and other similar studies from Uganda and China (17, 18). The reason might be more females are currently being engaged in risky sexual behaviors to cop-up with the growing living costs in the country, increased anatomical risks or might be associated with minimal women's decision role to have a safe sex (20).

Likewise, the proportion of recent infections is increased by nearly two folds among individuals with a recent history of commercial sex practice. This finding represents a true increase in HIV incidence among female sex workers and/or increased access to the targeted HIV testing service that had a high positive yield. In Ethiopia, even though it is unusual to get illicit drug users, this analysis revealed a 1.6% proportion of this risky social behaviors in the region. Therefore, emphasis should be given to such rare, hidden and protruding risky social behaviors that will further speed up the virus transmission, as this tiny information might be like the scenarios of the tip of the iceberg's phenomena or can be data entry errors at the health facility level. Also, in the final regression model, the variable illicit drug utilization is found to increase the proportion of recent infection nearly two folds, which is consistent with the finding in Germany (15). Inversely, the opportunity to detect recently infected HIV cases is decreased by half among clients who have contact with index cases as compared to their counterparts. The likely reason is a delay of primary and secondary contacts of index cases to get access to HIV testing services. Our study also confirms that the odds of recent infection are increased two folds among clients with secondary and above education as compared to clients without formal education. One would expect that this is the case; since as people get educated, they earn more and have more disposable income. This may lead to the financial ability to visit hotspots, consumption of alcoholic drinks and this may fuel engagement in risky behavior. Also, it might be linked to the situation that many of the literates are currently living around the towns where risky sexual networks and behaviors are prevalent, and especially the jobless may engage themselves to get income. This finding is in line with the finding from Tianjin of northern China (22).

Finally, it is mandatory for us to disclose the limitations of this analysis for readers. First, our finding is a proportion that might not represent the actual HIV incidence in the general population. However, the identified risk factors associated with recent infections can represent the actual phenomena in the source population. Another weakness in the methods is that viral load testing data are not available to further reclassify the HIV infections as confirmed recent (for non-suppressed) and long-term (for virally suppressed) infections.

## Conclusion and recommendations

In the Amhara region, the proportion of recent HIV infection is 14.2%; with marked sociodemographic variations. The risk factors associated with recent infection are female sex, increased education, commercial sex practice, no contact with index cases and illicit drug utilization. Therefore, we suggest to all HIV responders to target their HIV prevention efforts toward hot spot areas and sub-populations with high recent infection to prevent further transmission. Special emphasis should be given to case and network/cluster identification and investigation through an enhanced response. The success of the Enhanced Response can be maximized through a strong coordination platform like a Public Health Emergency Operation Center.

## Data availability statement

The raw data supporting the conclusions of this article will be made available by the authors, without undue reservation.

## Author contributions

All authors contributed significantly to the work reported, whether that is in the conception, study design, execution, acquisition of data, analysis and interpretation, or in all these areas, took part in drafting, revising, or critically reviewing the article, gave final approval of the version to be published, have agreed on the journal to which the article has been submitted, and agree to be accountable for all aspects of the work.

## Conflict of interest

The authors declare that the research was conducted in the absence of any commercial or financial relationships that could be construed as a potential conflict of interest.

## Publisher's note

All claims expressed in this article are solely those of the authors and do not necessarily represent those of their affiliated organizations, or those of the publisher, the editors and the reviewers. Any product that may be evaluated in this article, or claim that may be made by its manufacturer, is not guaranteed or endorsed by the publisher.

## Abbreviations

APHI: Amhara Public Health Institute
HIV: Human Immuno-Deficiency Virus
RedCap: Research data Capturing.

## References

[1] Federal HIV/AIDS Prevention and Control Office. HIV Prevention in Ethiopia: National Road Map. Addis Ababa: Federal HIV/AIDS Prevention and Control Office (2018). p. 1–43.

[2] World Health Organization. HIV/AIDS: Freamework for Action in the WHO Afican Region 2016-2016. Geneva: World Health Organization (2016). p. 1–11.

[3] World Health Organization. Consolidated Guidelines on Person-Centred HIV Strategic Information: Strengthening Routine Data for Impact. Geneva: World Health Organization (2022). Licence: CC BY-NC-SA 3.0 IGO.

[4] Ethiopian Public Health Institute. Guideline for HIV Case Based Surveillance in Ethiopia. Addis Ababa: Ethiopian Public Health Institute (2019).

[5] European Center for Disease Prevention and Control. Monitoring Recently Acquired HIV Infections in the European Context. Solna: European Center for Disease Prevention and Control (2013).

[6] ICAP at Columbia University. Tracking with Recency Assays to Control the Epidemic. https://icap.columbia.edu/wp-content/uploads/TRACE_Project_Brief.pdf (accessed November 01, 2022).

[7] Duval N Meredith G Delcher C Roussel B. Uniting and unifying existing HIV/AIDS case data to profile patient duplication, mobility and access to treatment and care services. in International AIDS Conference. (2012).

[8] Gerberding J. Centers for Disease Control and Prevention. Available online at: http://www.cdc.gov/hiv/pubs/070505_dearcolleague_gerberding.pdf (accessed August 16, 2022).

[9] PEPFAR Solutions Platform (BETA). Surveillance of Recent HIV Infections : Using a Point- of-Care Recency Test to Rapidly Detect and Respond to Recent Infections. Washinton, DC: PEPFAR Solutions Platform. Available online at: https://pepfarsolutions.squarespace.com/s/Surveillance-of-Recent-HIV-Infections_FINAL.pdf (accessed November 01, 2022).

[10] Yufenyuy EL Detorio M Dobbs T Patel HK Jackson K Vedapuri S. (2022) Performance evaluation of the asante rapid recency assay for verification of HIV diagnosis and detection of recent HIV-1 infections: Implications for epidemic control. PLOS Glob Pub Health. 2:e0000316. 10.1371/journal.pgph.0000316 PMC10021762 36962217

[11] SEDIA Bioscience Corporation. Available online at: https://www.sediabio.com/recency-resources/ (accessed September 20, 2022).

[12] FMOH Ethiopia. National Consolidated Guidelines for Comprehensive Hiv Prevention, Care and Treatment. Addis Ababa: Fmoh (2018). p. 1–238.

[13] *UNAIDS Issues New Fast-Track Strategy to End AIDS by 2030*. Available online at: https://www.pedaids.org/2014/11/20/unaids-issues-new-fast-track-strategy-to-end-aids-by-2030/ (accessed Auguest 31, 2021).

[14] *If You Take HIV Medicine to Know by How Much*. Available online at: https://www.cdc.gov/hiv/basics/livingwithhiv/protecting-others.html (accessed September 19, 2022).

[15] Hofmann A Hauser A Zimmermann R Santos-hövener C Bätzing-feigenbaum J Wildner S. Surveillance of recent HIV infections among newly diagnosed HIV cases in Germany between 2008 and 2014. BMC Infect Dis. (2017) 17:484. 10.1186/s12879-017-2585-4 28693564 PMC5504740

[16] Ang LW Low C Wong CS Boudville IC Paul M Sim H. Epidemiological factors associated with recent HIV infection among newly- diagnosed cases in Singapore, 2013 – 2017. BMC Pub Health. (2021) 21:430. 10.1186/s12889-021-10478-5 33653290 PMC7927232

[17] Mermin J Musinguzi J Opio A Kirungi W Ekwaru JP Downing R. Risk factors for recent HIV infection in Uganda. JAMA. (2008) 300. 10.1001/jama.300.5.540 18677026

[18] Chen M Ma Y Chen H Dai J Luo H Yang C. Demographic characteristics and spatial clusters of recent HIV-1 infections among newly diagnosed HIV-1 cases in Yunnan. BMC Pub Health. (2019) 19:1507 10.1186/s12889-019-7557-8 31711447 PMC6849305

[19] Welty S Motoku J Muriithi C Rice B Wit M De Ashanda B. Recent HIV infection surveillance in routine HIV testing in Nairobi, Kenya : a feasibility study. J Acquir Immune Defic Syndr. (2020) 84:5–9. 10.1097/QAI.0000000000002317 32058458

[20] Rwibasira GN Malamba SS Musengimana G Nkunda RCM Omolo J Remera E. Recent infections among individuals with a new HIV diagnosis in Rwanda, 2018-2020. PLoS ONE. (2021)16: 2018–20. 10.1371/journal.pone.0259708 34788323 PMC8598012

[21] Telford CT Tessema Z Msukwa M Arons MM Theu J Bangara FF. Geospatial transmission hotspots of recent HIV infection — Malawi, October 2019-March 2020. MMWR Recomm Rep. (2022) 71:329–34. 10.15585/mmwr.mm7109a1 35239633 PMC8893337

[22] Ning TL Guo Y Zheng MN. [The characteristics of recent HIV-1 infection and associated factors in Tianjin]. Zhonghua yu Fang yi xue za zhi. (2019) 53:323–6. 10.3760/cma.j.issn.0253-9624.2019.03 30841676

